# Integrin *α*3 is required for high‐frequency repetitive transcranial magnetic stimulation‐induced glutamatergic synaptic transmission in mice with ischemia

**DOI:** 10.1111/cns.14498

**Published:** 2023-10-23

**Authors:** Li Liu, Han Hu, Junfa Wu, Anthony J. Koleske, Hongting Chen, Nianhong Wang, Kewei Yu, Yi Wu, Xiao Xiao, Qun Zhang

**Affiliations:** ^1^ Department of Rehabilitation Medicine, Huashan Hospital Fudan University Shanghai China; ^2^ Behavioral and Cognitive Neuroscience Center Institute of Science and Technology for Brain‐Inspired Intelligence, Fudan University Shanghai China; ^3^ Departments of Molecular Biophysics and Biochemistry and Neuroscience Yale University New Haven Connecticut USA

**Keywords:** AMPAR, glutamatergic synaptic transmission, integrin *α*3, ischemic stroke, repetitive transcranial magnetic stimulation (rTMS)

## Abstract

**Background:**

Repetitive transcranial magnetic stimulation (rTMS) is an effective therapy in post‐stroke motor recovery. However, the underlying mechanisms of rTMS regulates long‐lasting changes with synaptic transmission and glutamate receptors function (including AMPARs or NMDARs) remains unclear.

**Methods:**

Mice were received 10‐Hz rTMS treatment once daily on the third day after photothrombotic (PT) stroke for 18 days. Motor behaviors and the Western blot were used to evaluate the therapeutic efficacy of 10‐Hz rTMS in the mice with PT model. Moreover, we used wild‐type (WT) and *NEX‐α3* ^*−/−*^ mice to further explore the 10‐Hz rTMS effect.

**Results:**

We found that 10‐Hz rTMS improved the post‐stroke motor performance in the PT mice. Moreover, the levels of AMPAR, vGlut1, and integrin *α*3 in the peri‐infarct were significantly increased in the rTMS group. In contrast, 10‐Hz rTMS did not induce these aforementioned effects in *NEX‐α3* ^*−/−*^ mice. The amplitude of AMPAR‐mediated miniature excitatory postsynaptic currents (EPSCs) and evoked EPSCs was increased in the WT + rTMS group, but did not change in *NEX‐α3* ^*−/−*^ mice with rTMS.

**Conclusions:**

In this study, 10‐Hz rTMS improved the glutamatergic synaptic transmission in the peri‐infract cortex through effects on integrin *α*3 and AMPARs, which resulted in motor function recovery after stroke.

## Graphical abstract

Model for integrin *α*3 involvement in the rTMS‐modulated glutamatergic synaptic transmission after PT stroke.

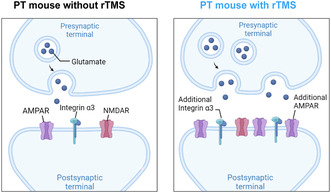

## 1 INTRODUCTION

Ischemic stroke is a major cause of disability and mortality worldwide.^1^ Estimates show that 70% of survivors have long‐term disability, which has a serious negative impact on their daily lives.^2^, ^3^ Other than traditional rehabilitation, new therapies, including nonpharmacological approaches, have gained attention to address the growing challenge of stroke and neurological disorders.

Repetitive transcranial magnetic stimulation (rTMS) is a promising therapy for patients with stroke.^4^ rTMS can modulate cortical excitability and increase neuronal plasticity. Specifically, high‐frequency rTMS (≥5 Hz) induces facilitatory effects, whereas low‐frequency rTMS (≤1 Hz) induces a reduction in synaptic efficiency.^5^ Clinical and animal investigations have both demonstrated that post‐stroke motor recovery can be effectively facilitated through the application of ipsilateral high‐frequency rTMS, which produces results similar to those seen with long‐term potentiation (LTP).^6^ However, the molecular mechanism underlying these effects remains elusive.

Glutamate transmission plays a fundamental role in LTP.^7^ Previous studies indicated that the 10‐Hz rTMS protocol could result in long‐lasting structural and functional changes at excitatory synapses by activating N‐methyl‐D‐aspartate receptors (NMDARs).^8^ Also, high‐frequency rTMS interferes with the postsynaptic accumulation of α‐amino‐3‐hydroxyl‐5‐methyl‐4‐isoxazolepropionate receptors (AMPARs), especially for GluA1 in an animal model of stroke.^9^ In addition, the TMS pulses induced the voltage‐sensitive current in the presynaptic compartment in an NMDAR‐independent manner as revealed by recording voltage‐clamp traces with glutamatergic blockers,^10^ while the long‐duration rTMS protocol increased the NMDAR‐dependent long‐term plasticity changes.^11^ Therefore, the complex mechanisms that mediate rTMS‐induced glutamatergic synaptic plasticity remain to be elucidated.

Integrins are a class of cell adhesion receptors expressed in excitatory neurons and glial cells. They are involved in synaptic maturation and plasticity as heterodimers (*αβ*).^12^ In mammals, 18 *α* and 8 *β* subunits can generate 24 different known heterodimers (*αβ*) with different binding properties and tissue distribution.^13^, ^14^ Our previous studies have shown integrin *α*3*β*1 is essential for synapse stability in the late postnatal mouse brain. The loss of integrin *α*3 from excitatory neurons causes reduced synapse densities in adult mice.^15^ Integrin *α*3 also influences NMDAR‐ and AMPAR‐mediated synaptic currents.^16^, ^17^ A reduction or loss of integrin *α*3 significantly decreases hippocampal synaptic plasticity and LTP, resulting in working memory impairment.^18^, ^19^ Above all, integrin *α*3 has a defined role in consolidating both structural and functional changes brought about by LTP. However, whether integrin *α*3 is involved in rTMS‐modulated synaptic plasticity after stroke is unclear.

This study aimed to investigate the role of integrin *α*3 in rTMS‐induced motor function recovery following stroke. First, we treated C57BL/6J mice with 10‐Hz rTMS for 18 days to observe motor recovery, glutamatergic synaptic transmission, and integrin *α*3 expression. We found that 10‐Hz rTMS could improve behavioral outcomes and increase the levels of AMPARs, glutamate transporters, and integrin *α*3 in the peri‐infarct region. Furthermore, we used mice bearing an excitatory neuron‐specific ablation of integrin *α*3 (*NEX‐α3* ^*−/−*^ mice) to establish a key role for integrin *α*3–AMPAR/NMDAR pathway in the effect of 10‐Hz rTMS. Compared to WT mice where 10‐Hz rTMS improved motor function and increased synaptic transmission following stroke, the same treatment did not improve outcomes in the *NEX‐a3* ^*−/−*^ mice. We also demonstrated the critical role of integrin *α*3 in AMPAR‐mediated excitatory synaptic current and strength increase effect induced by rTMS using the whole‐cell patch‐clamp recording. We concluded that the protective effects of 10‐Hz rTMS on post‐stroke mice required the integrin *α*3/AMPAR pathway.

## 2 MATERIALS AND METHODS

### 2.1 Animals

All procedures were approved by the Fudan University Animal Care and Use Committee (approval No. 2020 Huashan Hospital JS‐151) on March 31, 2020. Male C57BL/6J, *NEX‐α3* ^*−/−*^ ^15^ (conditional knockout integrin *α*3 in excitatory neurons in the cortex and hippocampus), and control WT mice, aged 6–8 weeks, were housed under standard laboratory conditions (12 h: 12 h light cycle) with controlled temperature (22 ± 2°C) and humidity (60%–70%) and allowed free access to food and water. *NEX‐α3* ^*−/−*^mice were generated by crossing germ‐line *itga3* ^20^ and *NEUROD6‐Cre /NEX‐Cre* ^21^ mice. The offspring were genotyped using polymerase chain reaction.

### 2.2 Photothrombotic stroke model

Mice were deeply anesthetized with sodium pentobarbital (50 mg/kg) intraperitoneally before the surgery, and a stereotactic apparatus was used to place animals. Photothrombotic (PT) stroke was induced in the left sensorimotor cortex (coordinates: 2.5–1.5 mm rostral to caudal and 0–4 mm lateral to the medial).^22^ Briefly, the hair was removed before the initial incision, and the skull was exposed. The mice were given 0.1% Rose Bengal (0.01 mL/g body weight,) by intravenous injection. Subsequently, a green laser beam (532 nm, ~50 mW intensity at the sample) was stereotactically positioned onto the target left sensorimotor cortex for 10 min.

### 2.3 Repetitive transcranial magnetic stimulation (rTMS) treatment

A magnetic stimulator (CCY‐II, Yiruide Medical Instrument Co., Ltd., Wuhan, China) was used to stimulate conscious mice on the third day after PT. Then, 10‐Hz repetitive transcranial magnetic stimulation (rTMS) at 35% maximum stimulator output (600 pulses per day) was administered for 18 consecutive days. During the treatment, the mouse heads were placed at the center of the circular coil.

### 2.4 Behavioral assessments

The rotarod test and the ladder rung walking test were used in this study. Mice were brought to the testing room for habituation 30 min prior to each test. All assessors were blinded to the group allocation.

#### 2.4.1 Rotarod test

The rotarod test was used to evaluate the coordination, balance, and motor performance of the mice.^23^ The test was performed on the 14th and 21st days post‐PT surgery. On the experimental day, the mice were placed on an accelerating rotating rod at a speed of 5–40 rpm for 5 min. The latencies to falling from the rod were recorded for each mouse, and the mean of the two fall latency values was used for data analysis. All animals received 3 days of training before the surgery.

#### 2.4.2 Ladder rung walking test

The ladder rung walking task was used to assess limb placement and limb coordination of the mice with stroke.^24^ The apparatus comprised two plexiglass side walls linked by inserting metal rungs (length: 100 cm, width: 5 cm, 30 cm above the ground). The test was conducted on the 14th and 21st days post‐PT after the pre‐surgery training sessions. Any deep paw slips, slight paw slips, replacement, and complete misses were scored as errors, and the forelimb slip rate was calculated as ratios of errors to total steps.

### 2.5 Western blot analysis

Mice were sacrificed on the last treatment day with rTMS. The peri‐infract cortex tissues (approximately 20 mg) were homogenized in 200 μL of RIPA lysis buffer (P0013B, Beyotime, Shanghai, China). Protein quantification was performed using a BCA protein assay kit (Melone Pharmaceutical, Dalian, China). Samples with 40 μg protein were separated by size using 10% sodium dodecyl sulfate–polyacrylamide gels and transferred onto nitrocellulose (NC) membranes. Membranes were blocked with 5% nonfat milk for 1 h at room temperature and then incubated overnight at 4°C with the following primary antibodies: integrin *α*3 (1:1000, 66,070–1, Proteintech), GluN2B (1:1000, 21,920‐1‐AP, Proteintech), *β*‐actin (1:1000, SD0034, SIMUWU), GluN2A (1:1000, 19,953‐1‐AP, Proteintech), GluA2/3/4 (1:1000, 2460, Cell Signaling), GluA1 (1:1000, 67,642‐1‐Ig, Proteintech), GAD65 (1:1000, 20,746‐1‐AP, Proteintech), and vGlut1 (1:1000, ab227805, Abcam). Membranes were washed with TBST and incubated with HRP‐conjugated goat anti‐mouse and goat anti‐rabbit antibody for 1 h at room temperature. ImageJ software was used to analyze protein bands.

### 2.6 Brain slice preparation and electrophysiological recording

Seven‐week‐old male mice were anesthetized with isoflurane and decapitated. Whole mouse brain was immediately put into an ice‐cold sucrose‐based cutting solution consisting of the following (in mM): 228 sucrose, 26 NaHCO_3_, 11 glucose, 2.5 KCl, 1 NaH_2_PO_4_, 7 MgSO_4,_ and 0.5 CaCl_2_, 330 mOsm, saturated with 95% O_2_ and 5% CO_2_, leading to the pH of 7.2–7.4. Coronary brain slices containing the peri‐infarct cortex region (300 μm thickness) were cut at 4°C using the cutting solution with a vibratome (VT 1200S) and then transferred to artificial cerebrospinal fluid (ACSF) containing the following (in mM): 119 NaCl, 26 NaHCO_3_, 11 glucose, 2.5 KCl, 1 NaH_2_PO_4_, 1.3 MgSO_4_, and 2.5 CaCl_2_, bubbled with 95% O_2_ and 5% CO_2_. Slices were kept at 30°C in the ACSF solution for 30 min and then at room temperature (22–24°C).

For whole‐cell recordings, we used a MultiClamp 700B Amplifier (Molecular Devices) to record pyramidal neurons in the peri‐infarct cortex region at room temperature. Neurons were observed using an infrared‐differential interference contrast microscope. Patch electrodes were drawn from thin‐walled glasses on a Sutter P97 puller to obtain an open‐tip resistance of 3–5 MΩ. A bipolar tungsten stimulating electrode was placed on the outer cortex to deliver evoked postsynaptic currents. AMPAR‐mediated excitatory postsynaptic currents (AMAPR‐EPSCs) were recorded in ACSF containing picrotoxin (PTX) in neurons in the voltage‐clamp mode at −70 mV. NMDAR‐EPSCs were recorded under the same condition as AMAPR‐EPSC recording but clamped at +40 mV. For postsynaptic current recordings, the patch electrodes were filled with a solution containing (in mM): 128 CsMeSO_4_, 10 HEPES, 3 ascorbic acid, 5 QX314 (lidocaine N‐ethyl bromide), 1 EGTA, 4 Na_2_ATP, 10 Na_2_CrePO_4_, 0.4 Na_3_GTP, and 4 MgCl_2_, pH adjusted to 7.2 with CsOH (290–295 mOsm). AMPAR‐mediated miniature excitatory postsynaptic current (mEPSC) was recorded with 1 mM tetrodotoxin (TTX) and 100 mM picrotoxin at −70 mV.

### 2.7 Statistical analysis

Electrophysiology raw‐trace offline analysis was performed using Igor 6.2 (WaveMetrics). EPSCs and mEPSCs were analyzed using Igor procedures, and incorrect mEPSC events were removed. Data were expressed as the mean ± standard error of the mean (SEM) and analyzed with Prism 8 software. The normal distribution of continuous data was detected by the Shapiro–Wilk test. One‐way analysis of variance (ANOVA) (one factor) or two‐way ANOVA (two factors) followed by Tukey's multiple comparison test was used to compare multiple groups. A *p* value <0.05 indicated a statistically significant difference.

## 3 RESULTS

### 3.1 rTMS promoted motor recovery after a photothrombotic stroke

**FIGURE 1:**
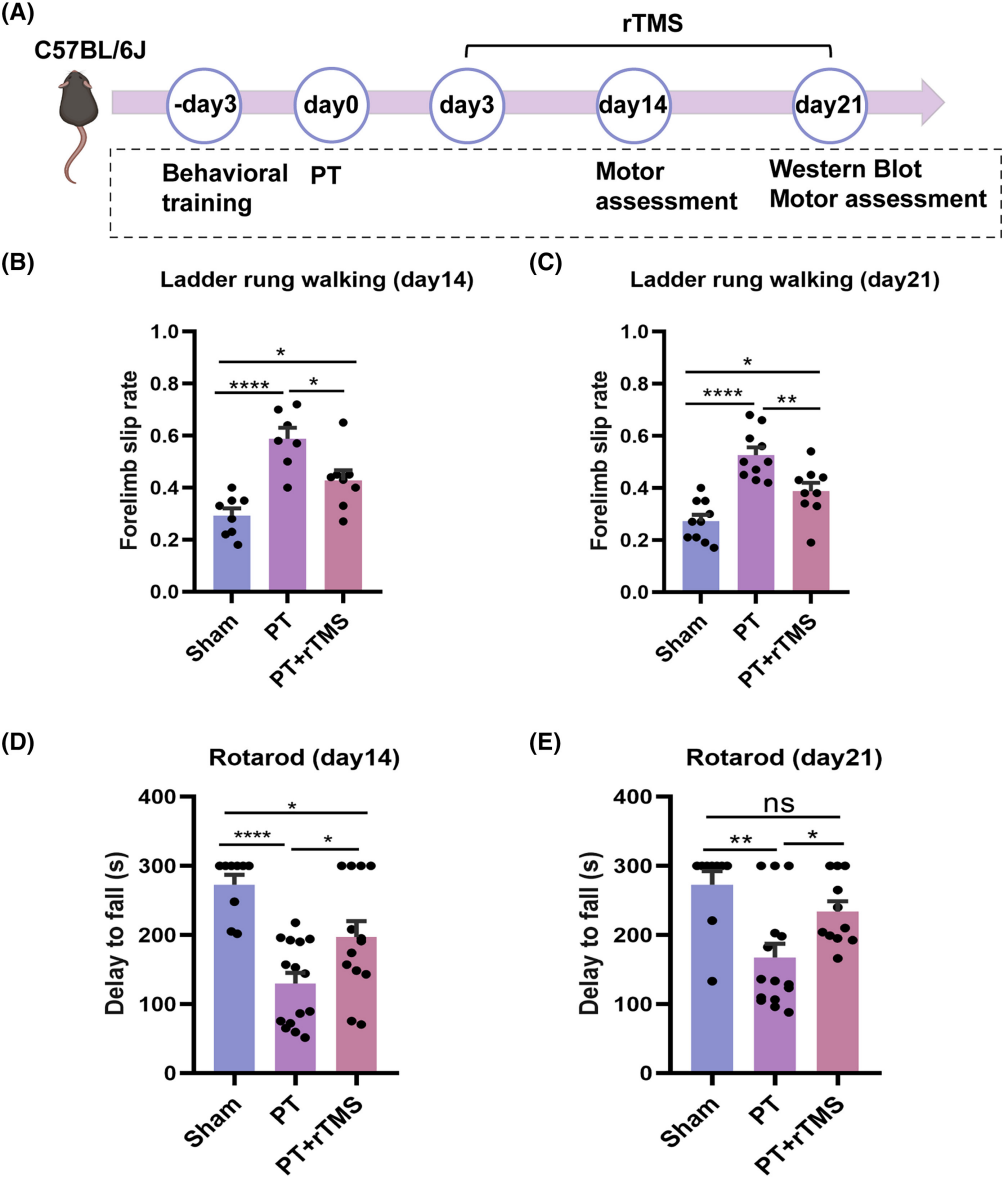
Effects of 10‐Hz rTMS on post‐stroke motor function recovery. (A) Time points of behavioral tests and surgery in C57/6 J mice. Behavioral training was started from the 3rd day before PT surgery, whereas motor assessments were conducted on the 14th and the 21st days after the PT stroke. rTMS was given from the 3rd to the 21st days post‐stroke. The mice were sacrificed on the 21st day after PT for Western blot. (B, C) Percentage of forelimb slips in the ladder rung walking test on the 14th and the 21st days after stroke in different groups. (D, E) Latency to fall off the rotarod on the 14th and the 21st days after stroke in different groups. Data are expressed as the mean ± SEM (*n* = 7–15). *p ≤ 0.05; **p ≤ 0.01; ***p ≤ 0.001; ****p ≤ 0.0001.

The ladder rung walking and rotarod tests were used to evaluate the motor functional recovery at different time points after PT to explore the therapeutic effects of 10‐Hz rTMS on post‐stroke mice (Figure 1A). In the ladder rung walking test (Figure 1B,C), a higher forelimb slip rate was observed on the 14th day after PT compared with that in the sham group (sham vs PT, *p* < 0.0001; sham vs PT + rTMS, *p* = 0.036). However, rTMS treatment decreased the forelimb slip rate (PT vs PT + rTMS, *p* = 0.016). Similarly, on the 21st day, the PT group (0.52 ± 0.02%) also exhibited more slips in the ladder rung walking test compared with the sham group (0.27 ± 0.02%), whereas the PT + rTMS groups showed fewer slips (0.38 ± 0.03%) (sham vs PT, *p* < 0.0001; sham vs PT + rTMS, *p* = 0.025; PT vs PT + rTMS, *p* = 0.006).

Additionally, in the rotarod test (Figure 1D,E), both PT and PT + rTMS results showed motor deficiency compared with that in the sham group on the 14th day post‐PT (sham vs PT, *p* < 0.0001; sham vs PT + rTMS, *p* = 0.003). However, motor deficiency only occurred in the PT group on the 21st day (sham vs PT, *p* = 0.0015). The PT + rTMS group showed a robust recovery, with no significant differences compared with the sham group (sham vs PT + rTMS, *p >* 0.05). The PT + rTMS group showed better performance on both the 14th (*p* = 0.02) and 21st (*p* = 0.03) days compared with the PT group.

### 3.2 Effects of rTMS on glutamatergic receptor and transporter levels in the peri‐infarct cortex

**FIGURE 2:**
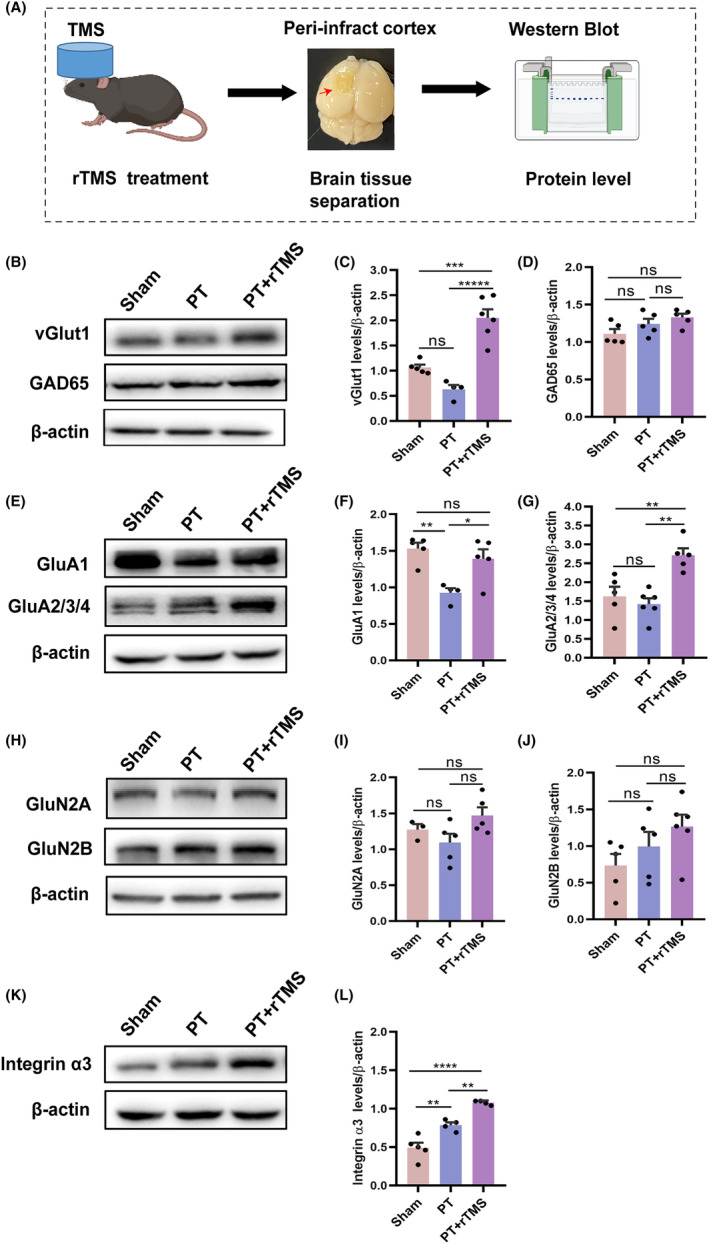
Effect of rTMS treatment on glutamatergic receptor and transporter levels in the peri‐infract cortex of mice with stroke. (A) Schematic representation of the tissue used for Western blot. (B) Representative blots of vGlut1 and GAD65 in different groups. (C, D) Quantitative analysis of vGlut1 and GAD65 levels. (E) Representative blot of GluA1 and GluA2/3/4 in different groups. (F,G) Quantitative analysis of GluA1 and GluA2/3/4 levels. (H) Representative blot of GluN2A and GluN2B in different groups. (I, J) Quantitative analysis of GluN2A and GluN2B levels. (K) Representative blot of integrin *α*3 in different groups. (L) Quantitative analysis of integrin *α*3 levels (*n* = 3–6). *p ≤ 0.05; **p ≤ 0.01; ***p ≤ 0.001; ****p ≤ 0.0001.

Given that high‐frequency rTMS (≥5 Hz) increased the cortical excitability,^25^ we detected the level of type 1 vesicle glutamate transporter (vGlut1) and glutamic acid decarboxylase 65 (GAD65) protein on the 21st day after rTMS treatment (Figure 2A). The level of vGlut1 increased significantly in the PT + rTMS group compared with the PT and sham groups (PT + rTMS vs sham, *p* = 0.0003; PT + rTMS vs PT, *p* < 0.0001; sham vs PT, *p* = 0.106, Figure 2B,C). No difference was found in GAD65 among the three groups (sham vs PT, *p* = 0.031; sham vs PT + rTMS, *p* = 0.06; PT + rTMS vs PT, *p* = 0.59, Figure 2D). We further measured the levels of different glutamatergic receptors, including the AMPAR subunits (GluA1 and GluA2/3/4) and the NMDAR subunits (GluN2A and GluN2B). As shown in Figure 2E, PT resulted in a decrease in the GluA1 compared with that in the sham group, (sham = 1.53 ± 0.07, PT = 0.92 ± 0.06, sham vs PT, *p* = 0.003), whereas rTMS significantly increased the level of GluA1 approximately to 1.39 ± 0.12 (sham vs PT + rTMS, *p* = 0.57; PT vs PT + rTMS, *p* = 0.019, Figure 2F). In addition, the levels of GluA2/3/4 were significantly different among the three groups. The PT + rTMS group had the highest levels, approximately 2.17 ± 0.18, but the levels in the sham and PT groups were 1.62 ± 0.25 and 1.42 ± 0.15, respectively (sham vs PT, *p* = 0.73; sham vs PT + rTMS, *p* = 0.005; PT vs PT + rTMS, *p* = 0.001, Figure 2G). For the GluN2A and GluN2B, no statistically significant difference was found among these groups (all *p >* 0.05), although the rTMS group showed a higher tendency (Figure 2H–J). In the PT group, the level of integrin *α*3 was elevated compared to the sham group (PT = 0.78 ± 0.03, sham = 0.49 ± 0.06); however, it remained notably lower than the levels observed in the rTMS group (PT + rTMS = 1.07 ± 0.01) (sham vs PT, *p* = 0.0043; sham vs PT + rTMS, *p* < 0.0001; PT vs PT + rTMS, *p* = 0.0064, Figure 2K,L).

### 3.3 Integrin *α*3 deletion inhibited the motor recovery effect of rTMS in post‐stroke mice

**FIGURE 3:**
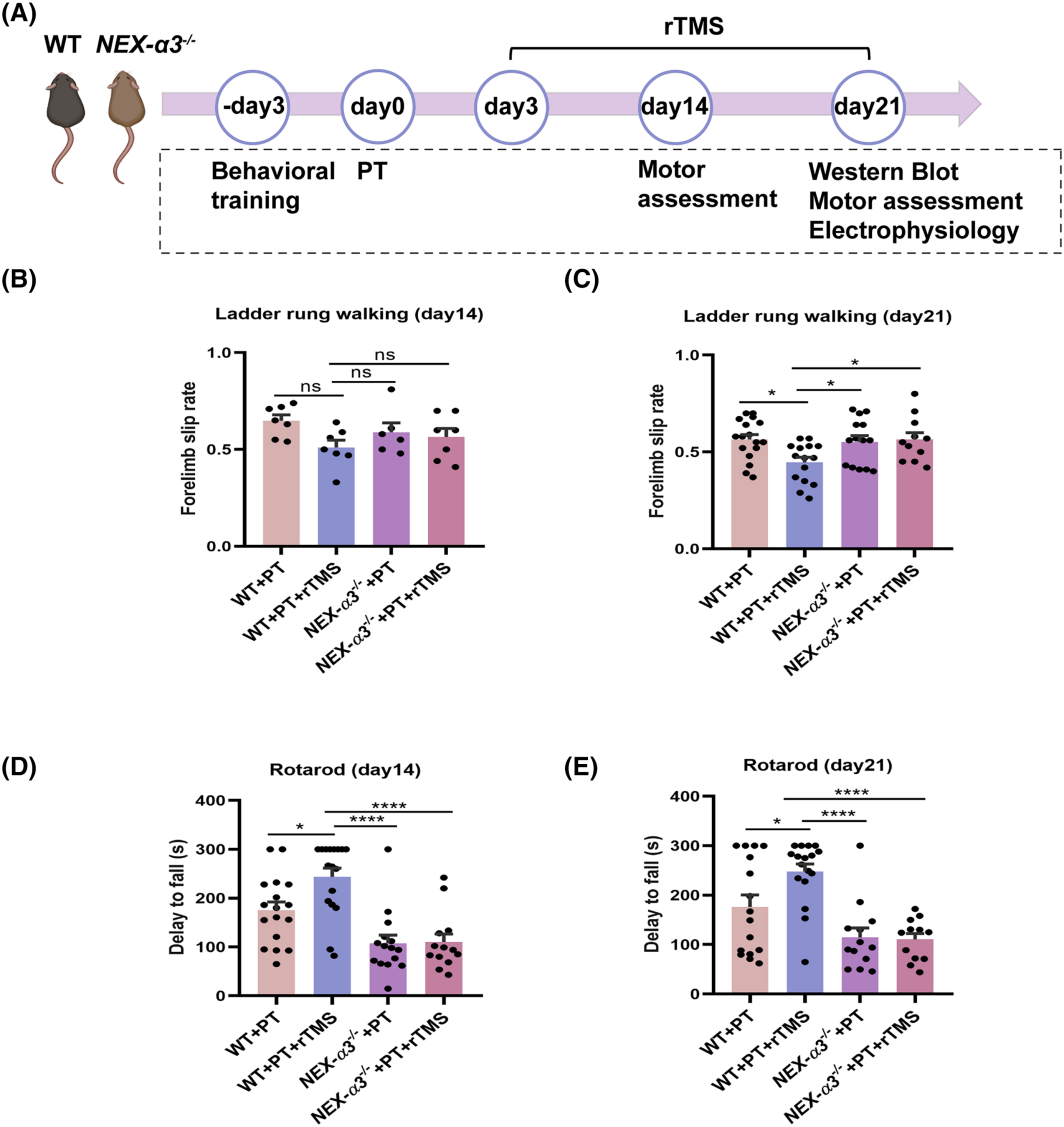
Effects of rTMS on motor recovery in WT and *NEX‐α3* ^*−/−*^ mice after PT. (A) Experimental process in WT and *NEX‐α3* ^*−/−*^ mice. Behavioral training was started from the 3rd day before PT, whereas the motor assessments were conducted on the 14th and 21st days after PT stroke. The rTMS was conducted from the 3rd day to the 21st day post‐stroke. The mice were sacrificed on the 21st day after PT for electrophysiological recording and Western blot. (B, C) Percentage of forelimb slips in the ladder rung walking test in different groups. (D, E) Latency to fall off the rotarod in different groups (*n* = 6–18). Data are expressed as the mean ± SEM. *p ≤ 0.05; **p ≤ 0.01; ***p ≤ 0.001; ****p ≤ 0.0001.

We analyzed the motor recovery in the WT + PT, *NEX‐α3* ^*−/−*^ + PT, WT + PT + rTMS, and *NEX‐α3* ^*−/−*^ + PT + rTMS groups on the 14th and 21st days post‐stroke to further explore the role of integrin *α*3 in the motor recovery effect of rTMS in post‐stroke mice (Figure 3A). In the ladder rung walking test (Figure 3B,C), no difference in the forelimb slip rate was detected in the four groups on the 14th day (WT + PT: 0.64 ± 0.03%; WT + PT + rTMS: 0.51 ± 0.03%; *NEX‐α3* ^*−/−*^ + PT: 0.58 ± 0.04%; *NEX‐α3* ^*−/−*^ + PT + rTMS: 0.58 ± 0.04%, all *p >* 0.05). On the 21st day, rTMS led to a decrease in the forelimb slip rate compared with the WT + PT group (WT + PT vs WT + PT + rTMS, *p* = 0.01), similar to the previous results shown in Figure 1. However, the protective effect of rTMS was abolished in *NEX‐α3* ^*−/−*^ mice (*NEX‐α3* ^*−/−*^ + PT vs *NEX‐α3* ^*−/−*^ + PT + rTMS, *p* = 0.93). Similarly, rTMS also significantly improved the stay time on the rotarod in the WT mice on both the 14th (*p* = 0.025) and 21st (*p* = 0.02) days post‐stroke, while no difference was found in the *NEX‐α3* ^*−/−*^ mice (*NEX‐α3* ^*−/−*^ + PT vs *NEX‐α3* ^*−/−*^ + PT + rTMS, *p* = 0.999 and *p* = 0.998) on the 14th and 21st days post‐stroke (Figure 3D,E).

### 3.4 Integrin α3 deletion inhibited the effects of rTMS on post‐stroke glutamatergic receptor and transporter levels

**FIGURE 4:**
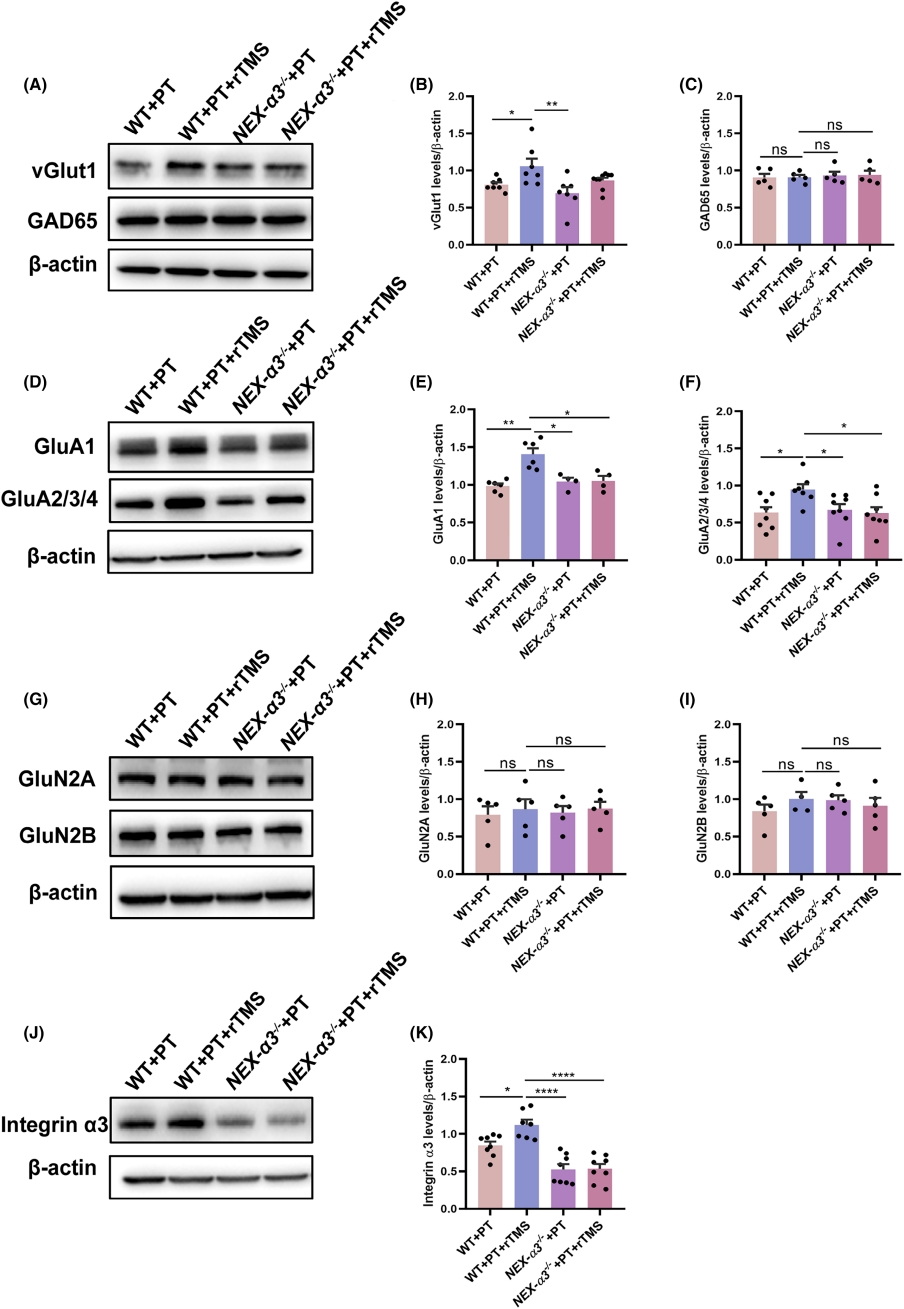
Effects of rTMS treatment on glutamatergic receptor and transporter levels in WT and *NEX‐α3* ^*−/−*^ mice after PT. (A) Representative blot of vGlut1 and GAD65 in different groups. (B, C) Quantitative analysis of vGlut1 and GAD65 levels. (D) Representative blot of GluA1 and GluA2/3/4 in different groups. (E, F) Quantitative analysis of GluA1 and GluA2/3/4 levels. (G) Representative blot of GluN2A and GluN2B in different groups. (H, I) Quantitative analysis of GluN2A and GluN2B levels. (J) Representative blot of integrin *α*3 in different groups. (K) Quantitative analysis of integrin *α*3 levels. Data are expressed as the mean ± SEM (*n* = 4–8). *p ≤ 0.05; **p ≤ 0.01; ***p ≤ 0.001; ****p ≤ 0.0001.

Integrin α3 levels in the *NEX‐α3* ^*−/−*^ + PT and *NEX‐α3* ^*−/−*^ + PT + rTMS groups were lower than that in the WT + PT and WT + PT + rTMS groups in the peri‐infract cortex (WT + PT vs *NEX‐α3* ^*−/−*^ + PT, *p* = 0.005, WT + PT + rTMS vs *NEX‐α3* ^*−/−*^ + PT + rTMS, *p* < 0.0001), whereas the rTMS increased the integrin *α*3 levels in the WT groups (*p* = 0.03, Figure 4J,K), similar to the previous data in Figure 2. No difference in GAD 65 was observed across the four groups (Figure 4C, all *p >* 0.05). However, an increase in vGlut1 was observed in the WT + PT + rTMS group compared with the WT + PT group (*p* = 0.039), whereas no difference was found between the *NEX‐α3* ^*−/−*^ + PT and *NEX‐α3* ^*−/−*^ + PT + rTMS groups (*p* = 0.30) (Figure 4A,B). Moreover, the levels of GluN2A and GluN2B were almost the same among the four groups (Figure 4G–I, all *p >* 0.05), but GluA1 and GluA2/3/4 levels significantly increased in the WT + PT + rTMS group compared with the WT + PT and *NEX‐α3* ^*−/−*^ + PT + rTMS groups (Figure 4D) (GluA1: WT + PT vs WT + PT + rTMS, *p* = 0.001; WT + PT + rTMS vs *NEX‐α3* ^*−/−*^ + PT + rTMS, *p* = 0.023; GluA2/3/4: WT + PT vs WT + PT + rTMS, *p* = 0.0108; WT + PT + rTMS vs *NEX‐α3* ^*−/−*^ + PT + rTMS, *p* = 0.0089). With integrin α3 deletion, rTMS had no significant effect on GluA1 and GluA2/3/4 levels (Figure 4E,F *p >* 0.05).

### 3.5 Integrin *α*3 deletion inhibited the increase in post‐stroke excitatory synaptic transmission by rTMS

**FIGURE 5:**
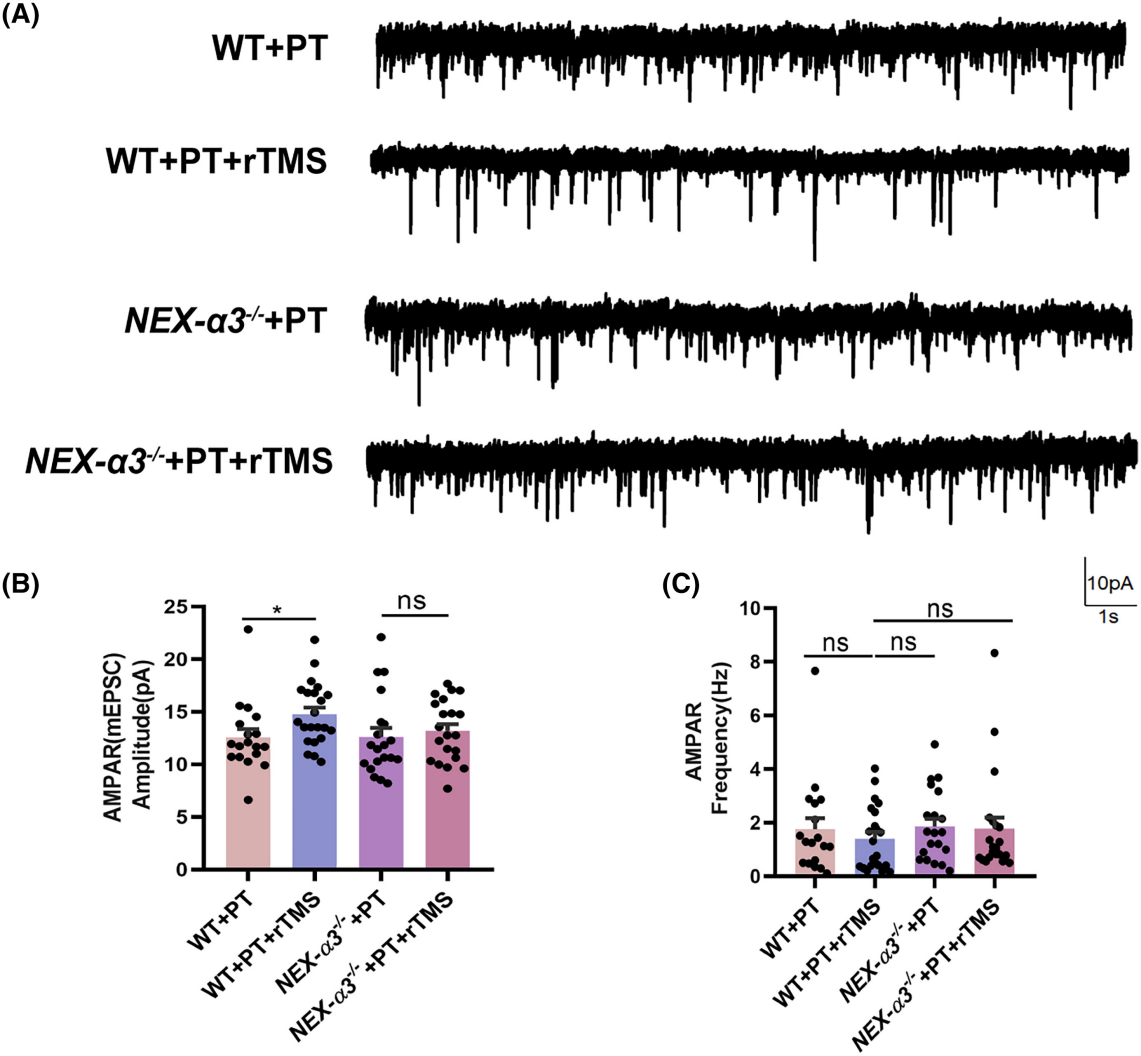
Effects of rTMS treatment on mEPSCs in WT and *NEX‐α3* ^*−/−*^ mice after PT. (A) Representative traces of the AMPAR‐mEPSCs in different groups. (B) Quantitative analysis of the amplitude of AMPAR‐mEPSCs. (C) Quantitative analysis of the frequency of AMPAR‐mEPSCs. Data are expressed as the mean ± SEM (*n* = 18–22). *p ≤ 0.05.

**FIGURE 6:**
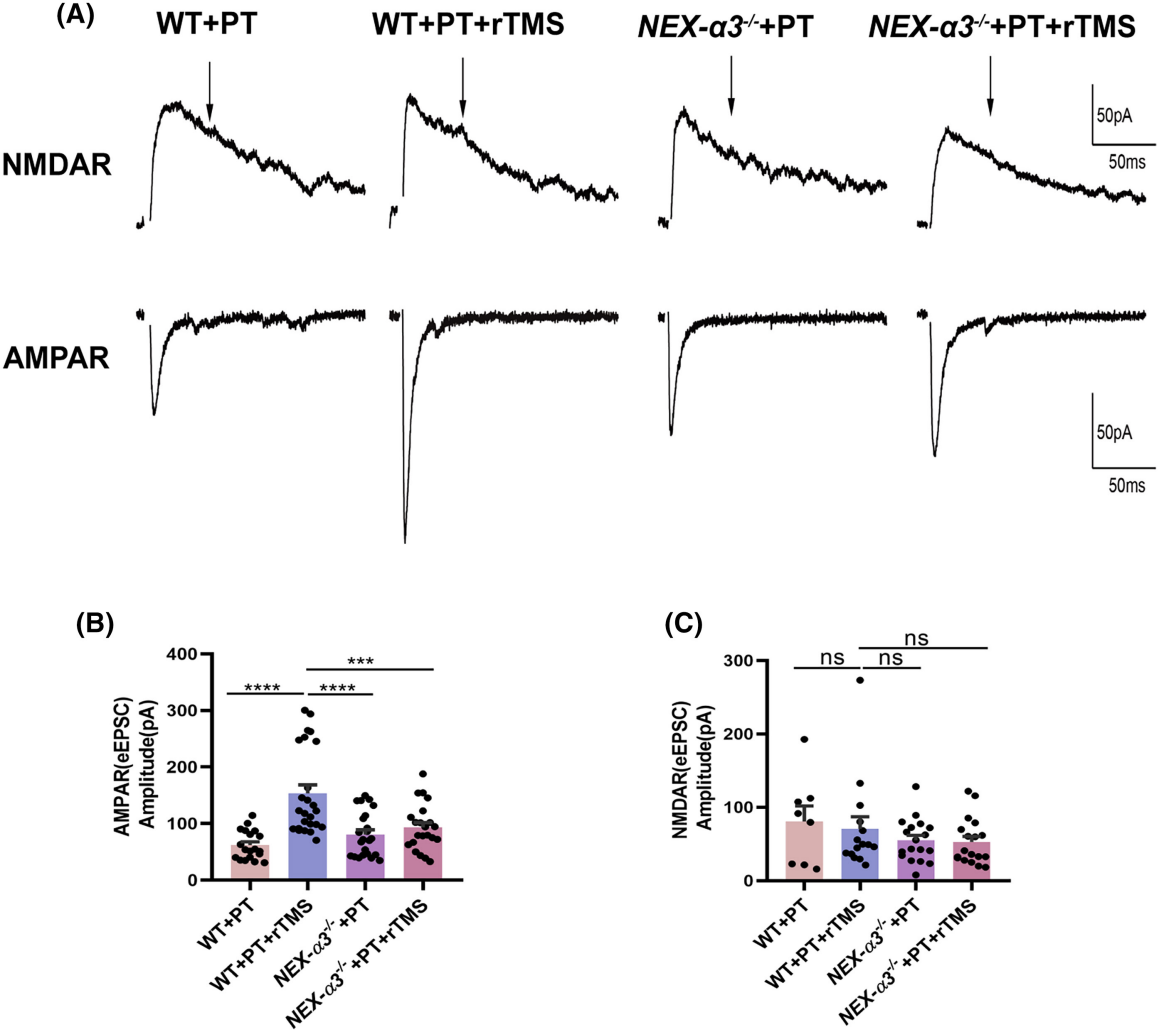
Effects of rTMS treatment on the evoked EPSCs in WT and *NEX‐α3* ^*−/−*^ mice after PT. (A) Representative traces of AMPAR and NMDAR currents in different groups. (B, C) Statistical analysis of the amplitude of AMPAR‐eEPSCs and NMDAR‐eEPSCs. Data are expressed as the mean ± SEM (*n* = 8–25). ***p ≤ 0.001; ****p ≤ 0.0001.

We used whole‐cell patch‐clamp recording to analyze the properties of mEPSCs in the peri‐infract cortex neurons in different treatment groups to identify the mechanisms underlying the increase in excitatory synaptic transmission by rTMS. On the 21st day after PT, rTMS led to a significant increase in the AMPAR‐mediated miniature excitatory postsynaptic current (mEPSC) amplitude compared with the non‐stimulated groups (WT + PT: 12.57 ± 0.41; WT + PT + rTMS: 14.77 ± 0.63; *p* = 0.01), whereas no significant difference in effect was detected between the *NEX‐α3* ^*−/−*^ + PT and *NEX‐α3* ^*−/−*^ + PT + rTMS groups (*NEX‐α3* ^*−/−*^ + PT: 12.60 ± 0.85, *NEX‐α3* ^*−/−*^ + PT + rTMS: 13.18 ± 0.64, *p* = 0.84). In contrast, the frequency of AMPAR‐mediated mEPSCs was not significantly different in the four groups (WT + PT: 1.75 ± 0.41; WT + PT + rTMS: 1.39 ± 0.25; *NEX‐α3* ^*−/−*^ + PT: 1.85 ± 0.29; *NEX‐α3* ^*−/−*^ + PT + rTMS: 1.77 ± 0.42; all *p >* 0.05, Figure 5A–C). To further explore excitatory synaptic strength, we measured the AMPAR‐ and NMDAR‐mediated evoked EPSCs in the same neurons by voltage clamping at –70 mV and + 40 mV, respectively. rTMS significantly increased the AMPAR‐mediated currents without affecting NMDAR‐mediated currents in the WT + PT group. The average amplitude of AMPAR‐ and NMDAR‐mediated currents was 61.80 ± 5.86 and 80.53 ± 21.29, respectively, in the WT + PT group. After the application of rTMS, the average amplitude of AMPAR‐ and NMDAR‐mediated currents in the WT + PT + rTMS group was 153.41 ± 15.19 and 70.73 ± 16.36, respectively. rTMS only increased the average amplitude of the AMPAR‐mediated current (WT + PT vs WT + PT + rTMS: *p* < 0.0001), without influencing the NMDAR‐mediated current (*p* = 0.96). However, not only the NMDAR‐mediated current, rTMS also did not change the average amplitude of the AMPAR‐mediated current in the *NEX‐α3* ^*−/−*^ + PT + rTMS group (*NEX‐α3* ^*−/−*^ + PT vs *NEX‐α3* ^*−/−*^ + PT + rTMS, *p* = 0.99 and *p* = 0.83, Figure 6A–C).

## 4 DISCUSSION

This study has revealed that a course of 10‐Hz rTMS treatment spanning 18 days not only ameliorated motor recovery in post‐stroke mice but also instigated an enhancement in glutamatergic synaptic transmission, alongside elevated levels of AMPARs, vGlut1, and integrin *α*3. Notably, these changes were paralleled by augmented AMPAR‐mediated currents, as observed through assessments of both AMPAR‐mediated mEPSCs and eEPSCs. Intriguingly, no discernible disparity in post‐stroke motor recovery emerged between mice subjected to rTMS and those without it, particularly evident in cases involving *NEX‐α3* ^*−/−*^ mice. From these observations, we deduced that the positive impact of 10‐Hz rTMS on motor recovery among post‐stroke mice potentially hinges upon the regulation of the integrin *α*3/AMPAR signaling pathway. Different from the earlier studies that rTMS promotes synaptic plasticity by upregulating AMPARs post‐stroke,^26^ our findings provide direct evidence that integrin *α*3 is a critical factor in rTMS‐mediated AMPAR upregulation. This suggests that the direct modulation of integrin *α*3 may offer novel avenues to mitigate stroke‐related dysfunction, via the regulation of glutamatergic neurotransmission.

In this study, we found that the induced photothrombosis triggered an increase in the expression of GAD65, coupled with a concomitant decrease in the expression of the glutamatergic transporter vGlut1 within the peri‐infarct cortex. In contrast, administration of 10‐Hz rTMS resulted in notable elevations in the levels of vGlut1, GluA1, and GluA2/3/4. Although there was a slight increment in the levels of GluN2A and GluN2B following 10‐Hz rTMS, these distinctions did not attain statistical significance. These changes in protein expression are likely tied to a reduction in cortical excitability post‐stroke. In addition, the level of integrin *α*3 in the PT group was elevated compared to the sham group; however, it remained notably lower than the levels observed in the rTMS group. Considering that integrin *α*3 plays a role in the cellular membrane localization of AMPARs, we hypothesized that this relationship might follow a dose‐dependent pattern. The heightened integrin *α*3 expression in the PT group could potentially signify a compensatory response, albeit insufficient to induce significant changes in AMPARs at this stage. Nevertheless, further investigations are imperative to elucidate this divergence.

Aligned with the alterations in the glutamatergic signaling components, a discernible rise in the amplitude of AMPAR‐mediated mEPSCs and eEPSCs emerged in post‐stroke mice subjected to rTMS. These findings harmonize with existing evidence that high‐frequency rTMS heightens GluA1 levels and promotes the insertion of AMPARs into synapses. However, prior investigations have positioned NMDARs and AMPARs as the principal receptors underpinning rTMS effects,^27^ the involvement of NMDARs in rTMS‐induced neuronal plasticity remains a possibility. Yet, the amplitudes of NMDAR‐mediated mEPSCs and eEPSCs remained unperturbed in our results. This hints at a preference for 10‐Hz rTMS to primarily activate rapidly and briefly opening AMPARs, which could interact with NMDARs characterized by slower depolarizing currents. Thus, more extensive rTMS protocols could illuminate the role of NMDA receptor‐mediated long‐term plasticity in future inquiries.^28^, ^29^

Building upon our prior previous studies highlighting integrin *α*3's role in synaptic maturation, plasticity, and neuronal stability,^15^ we found an upsurge in integrin *α*3 expression within the peri‐infarct cortex following a stroke event. Furthermore, we have established that integrin *α*3 plays a pivotal role in rTMS‐induced functional recovery. Notably, the beneficial effects of rTMS on motor recovery were nullified with the loss of the integrin *α*3 gene in excitatory neurons, accompanied by an absence of rTMS impact on AMPAR‐mediated currents and GluA1 and GluA2/3/4 clustering in *NEX‐α3* ^*−/−*^ mice. Considering that rTMS could modulate the neuronal activity in the ipsilateral hemisphere, it is plausible for integrin *α*3, pivotal in cell adhesion and migration, to transduce activity‐dependent signals leading to AMPAR and vGlut1 recruitment and insertion. Furthermore, since the integrin *α*3‐regulated Arg kinase coordinates the maturation of presynaptic and postsynaptic compartments within a subset of hippocampal synapses,^30^ this process aligns with the significance of LTP‐like plasticity in the plastic changes induced by rTMS. Consequently, it is conceivable that integrin *α*3's response to rTMS encompasses multifaceted pathways. However, the precise interplay between AMPAR activation and integrin *α*3‐mediated signaling in rTMS‐induced post‐stroke recovery necessitates further investigation.

Some limitations were existed in this study. Since the integrin *α*3 is an accomplice of integrin *β*1, the integrin *α*3*β*1 ligands exert influence on neuronal development, structure, regeneration, maintenance, and plasticity.^31^, ^32^ Nonetheless, our study did not explore the role of integrin *β*1 in rTMS‐induced motor recovery post‐stroke. Furthermore, during the course of this research, we concurrently pursued two investigations linked to integrin *α*3, involving distinct cohorts of mice subjected to behavioral tests and protein assessments across various time points. The omission of time‐variable analyses could potentially limit our ability to fully grasp the evolving behavioral changes following stroke in different groups. Lastly, the intricate interactions between extracellular matrix (ECM) proteins^33^ and integrins post‐ischemic stroke need more investigation. For instance, *β*1 integrin in conjunction with laminin provides scaffolds, guiding neural migration toward injury sites post‐stroke.^34^ Our prior study demonstrated the functional interaction between laminin *α*5 and integrin *α*3 in regulating dendritic spine density and animal behavior.^35^ The broader impact of integrin–extracellular matrix remodeling following rTMS treatment in stroke remains a fascinating area for exploration, considering its potential significance for neurogenesis and angiogenesis.

## 5 CONCLUSIONS

Our study showed that 10‐Hz rTMS promoted the glutamatergic synaptic transmission after stroke. Additionally, we found that integrin *α*3 is critical for AMPAR‐dependent motor functional improvement induced by 10‐Hz rTMS. These results might highlight integrin *α*3 as a potential novel therapeutic target for treating stroke.

## AUTHOR CONTRIBUTIONS

Yi Wu, Xiao Xiao, Junfa Wu, Nianhong Wang, and Qun Zhang designed the study and supervised the entire project. Anthony J. Koleske provided the *NEX‐α3* ^*−/−*^ mice. Li Liu, Han Hu, Hongting Chen, and Kewei Yu performed the experiments and analyzed the data. Li Liu, Qun Zhang, Xiao Xiao, and Anthony J. Koleske wrote the manuscript.

## CONFLICT OF INTEREST STATEMENT

The authors have no conflicts of interest to declare.

## Supporting information


Data S1

## Data Availability

The data that support the findings of this study are available from the corresponding author upon reasonable request.
