# Supplementary material for: Integrin α3 is required for high‐frequency repetitive transcranial magnetic stimulation‐induced glutamatergic synaptic transmission in mice with ischemia
Source: CNS Neurosci Ther. 2023 Oct 23;30(4):e14498. doi: 10.1111/cns.14498 (PMC11017422; doi:10.1111/cns.14498)
Supplement: Supplementary file 1 — Data S1 [file CNS-30-e14498-s001.pdf]

Dear Editor, we apologized for the negligence in the Western Blot test.We did not mark the position of the molecular weight standard during membrane exposed. In our experiment, we concurrently pursued two investigations linked to integrin α3, involving distinct cohorts of mice subjected to protein assessments across various time points.In addition, sometimes experiments fail due to various factors, so there are different dot numbers of each group. Meanwhile,the grouping of the original blot images were also inconsistent.We are very sorry for this, and we will pay attention to it in the future research.

The original and uncropped blot images in **Figure 2** are shown below and the bolts marked by the red frame are those that appear in the manuscript.

Full unedited blot for Figure 2E

GluA2/3/4 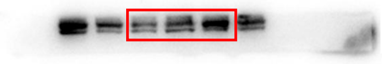 100KD

Full unedited blot for Figure 2B

vGlut1 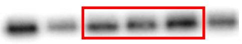 62KD

β-actin 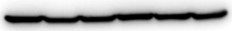 42KD

Full unedited blot for Figure 2H

GluN2B 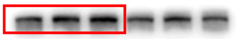 166KD

Full unedited blot for Figure 2E

GluA1 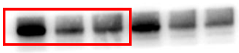 100KD

Full unedited blot for Figure 2E

β-actin 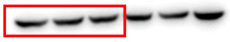 42KD

Full unedited blot for Figure 2H

GluN2A 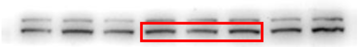 160KD

Full unedited blot for Figure 2H

β-actin 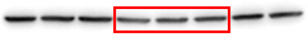 42KD

Full unedited blot for Figure 2B

GAD65 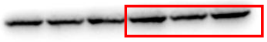 65KD

Full unedited blot for Figure 2B

β-actin 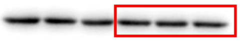 42KD

Full unedited blot for Figure 2K

Integrin α3 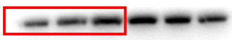 120KD

Full unedited blot for Figure 2K

β-actin 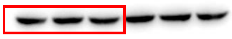 42KD

The original and uncropped blot images in **Figure 4** are shown below and the bolts marked by the red frame are those that appear in the manuscript.

Full unedited blot for Figure 4A

GAD65 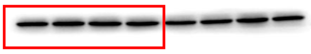 65KD

Full unedited blot for Figure 4A

β-actin 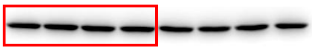 42KD

Full unedited blot for Figure 4D

GluA1 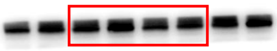 100KD

Full unedited blot for Figure 4A

vGlut1 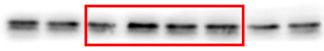 62KD

β-actin 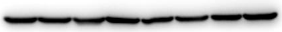 42KD

Full unedited blot for Figure 4J

Integrin α3 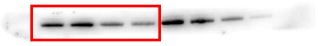 120KD

Full unedited blot for Figure 4J

β-actin 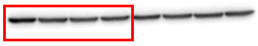 42KD

Full unedited blot for Figure 4G

GluN2B 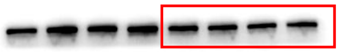 166KD

Full unedited blot for Figure 4D

GluA2/3/4 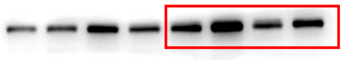 100KD

Full unedited blot for Figure 4D

β-actin 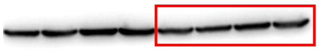 42KD

Full unedited blot for Figure 4G

GluN2A 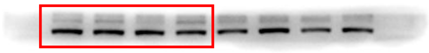 160KD

Full unedited blot for Figure 4G

β-actin 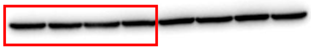 42KD
